# Locking up the AS1411 Aptamer with a Flanking Duplex: Towards an Improved Nucleolin-Targeting

**DOI:** 10.3390/ph14020121

**Published:** 2021-02-04

**Authors:** André Miranda, Tiago Santos, Eric Largy, Carla Cruz

**Affiliations:** 1 CICS-UBI—Centro de Investigação em Ciências da Saúde, Universidade da Beira Interior, Av. Infante D. Henrique, 6200-506 Covilhã, Portugal; andre.miranda@ubi.pt (A.M.); tiagoaasantos@hotmail.com (T.S.); 2 Laboratoire Acides Nucléiques: Régulations Naturelle et Artificielle, Université de Bordeaux, INSERM & CNRS, (ARNA, U1212, UMR5320), IECB, 2 rue Robert Escarpit, 33607 Pessac, France; eric.largy@u-bordeaux.fr

**Keywords:** AS1411 derivative, G-quadruplex, DNA aptamers, biophysical characterization

## Abstract

We have designed AS1411-N6, a derivative of the nucleolin (NCL)-binding aptamer AS1411, by adding six nucleotides to the 5′-end that are complementary to nucleotides at the 3′-end forcing it into a stem-loop structure. We evaluated by several biophysical techniques if AS1411-N6 can adopt one or more conformations, one of which allows NCL binding. We found a decrease of polymorphism of G-quadruplex (G4)-forming sequences comparing to AS1411 and the G4 formation in presence of K^+^ promotes the duplex folding. We also studied the binding properties of ligands TMPyP4, PhenDC3, PDS, 360A, and BRACO-19 in terms of stability, binding, topology maintenance of AS1411-N6, and NCL recognition. The melting experiments revealed promising stabilizer effects of PhenDC3, 360A, and TMPyP4, and the affinity calculations showed that 360A is the most prominent affinity ligand for AS1411-N6 and AS1411. The affinity determined between AS1411-N6 and NCL denoting a strong interaction and complex formation was assessed by PAGE in which the electrophoretic profile of AS1411-N6 showed bands of the dimeric form in the presence of the ligands and NCL.

## 1. Introduction

Aptamers are a class of oligonucleotides (RNA or DNA) that can recognize and bind their targets (proteins, cells, bacteria, and viruses). Their binding affinities range from *K*_D_ of pM to µM and are generated by an iterative screening process of complex oligonucleotide libraries employing a process termed by systemic evolution of ligands by exponential enrichment, or simply SELEX [1,2]. The aptamers formed from G-rich sequences, besides recognizing the target, are capable of folding into G-quadruplexes (G4) structures and take out advantages (chemical and thermodynamic stability, nucleases resistance, low immunogenicity, and enhanced cellular uptake) [3,4].

An interesting G-rich aptamer is AS1411, which has been reported as an aptamer of nucleolin (NCL) and studied for cancer therapy and diagnosis [5,6,7,8]. NCL is a protein overexpressed at the surface of several cancer cells and it is related with cell growth, proliferation, and survival [6,9]. AS1411 has demonstrated a remarkable anticancer activity and binds with high affinity to cell surface NCL. In order to improve its potential and features, several modifications have been made to decrease the polymorphism of this aptamer. Indeed, despite the broad applicability of AS1411, its conformational polymorphism limits the characterization of its structural features, namely the resolution of 3D structures. A previous study developed by Dailey et al. identified eight different conformers of AS1411 under the same experimental conditions [10]. To overcome this structural drawback, researchers successfully isolated a single structural conformation of AS1411 by modifying the original sequence [11].

**Figure 1:**
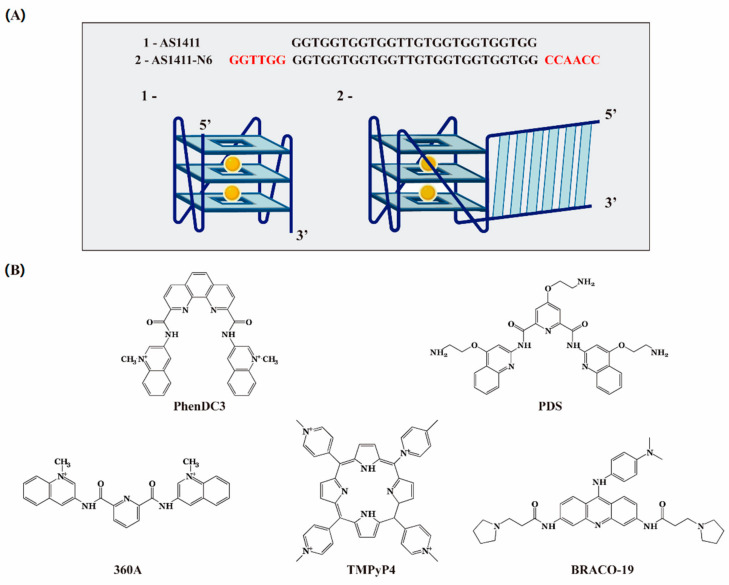
(**A**) Oligonucleotides sequences and a possible representation of the duplex/G4 (AS1411-N6) and aptamer (AS1411). The aptamer derivative was termed as AS1411-N6 due to the six nucleotides portion added to both ends. The structures are merely representative and does not consider the actual topology and orientation of bases. (**B**) Chemical structures of G4 ligands. Chosen in this study were distinct classes of ligands: acridine (BRACO-19); pyridines (PDS and 360A); phenanthroline (PhenDC3) and porphyrin (TMPyP4).

Based on these considerations, we hypothesized that it would be possible to decrease the conformational polymorphism by retaining the original sequence but adding short 5′ and 3′ flanking sequences capable of hybridizing with one another. The modified aptamer, denominated by AS1411-N6, is presented in Figure 1A. Furthermore, we hypothesized that this duplex addition can confer the property of an aptamer beacon in the presence of NCL, if suitably fluorophore labeled. Aptamer beacons combine the advantages of aptamers and the signal transduction of molecular beacons [12]. There are different strategies to design molecular beacons [13], but a similar “light-up” strategy was employed by Hamaguchi et al. in the design of G4 aptamers for the detection of thrombin protein [12].

This work is divided into two parts: firstly, we evaluate the effect of increasing K^+^ concentration on the G4 and duplex formation, stability, and kinetics in AS1411-N6, always comparing to AS1411 (Figure 1A). In the second part, we used well-known G4s ligands TMPyP4, PhenDC3, PDS, 360A, and BRACO-19 (Figure 1B), to improve G4 binding and stabilization in AS1411-N6, and evaluate if the complexes AS1411-N6/ligands improve binding to NCL. For this purpose, we employ circular dichroism (CD), UV absorption, Nuclear Magnetic Resonance (NMR), fluorescence titrations, fluorescence resonance energy transfer (FRET) melting, surface plasmon resonance (SPR) biosensor, and non-denaturing polyacrylamide gel electrophoresis (PAGE). 

## 2. Results and Discussion

The AS1411 is an NCL-binding aptamer capable to form G4 structure and presents a higher degree of polymorphism [3,10]. For this reason, it is difficult to determine the biologically relevant structure(s) and define its three-dimensional structure. Previous studies found that the majority of conformations display the classical parallel G4 [6]. Due to the potential of the AS1411 aptamer, several modifications have been made to improve its polymorphism [11].

Based on these considerations, we designed a derivative of AS1411 termed AS1411-N6 by adding six nucleotides to the 5′-end that are complementary to nucleotides at the 3′-end of the oligonucleotide. We evaluated the G4 formation in AS1411-N6, by changing the salt concentration, adding G4 ligands and determining NCL binding using several techniques such as circular dichroism (CD), UV absorption, Nuclear Magnetic Resonance (NMR), fluorescence titrations, fluorescence resonance energy transfer (FRET) melting, surface plasmon resonance (SPR) biosensor, and non-denaturing polyacrylamide gel electrophoresis (PAGE) analysis.

The spectroscopic properties and conformational behaviors of AS1411-N6 were first investigated by CD spectroscopy in comparison with AS1411. The stabilization effect of KCl in the G4 formation and topology was evaluated by CD titrations.

**Figure 2:**
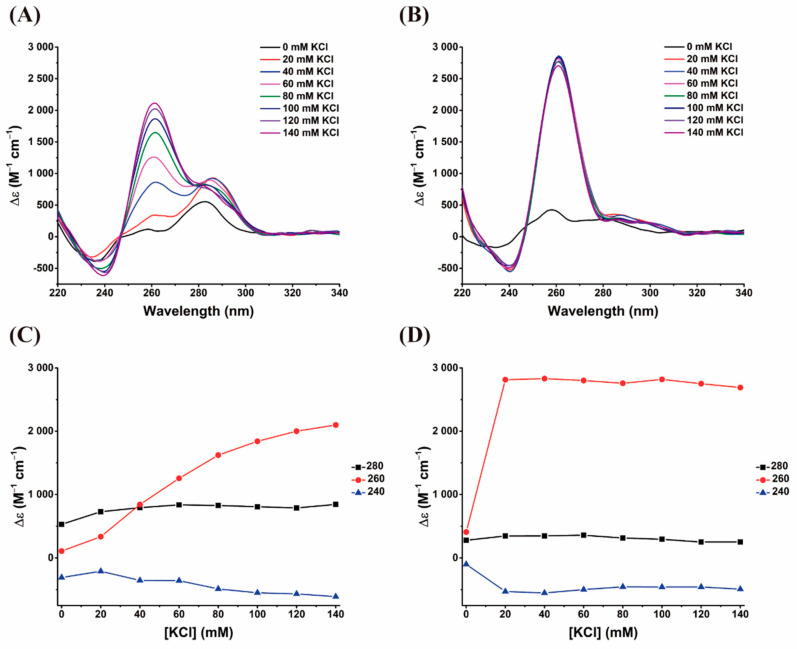
CD titration of 10 μM (**A**) AS1411-N6 and (**B**) AS1411 solutions containing K^+^ (20–140 mM range). (**C**) AS1411-N6 and (**D**) AS1411 correspond to the plots of the potassium-dependent evolution of the three characteristic bands (240, 260, 280 nm).

The CD spectra of AS1411-N6 and AS1411 are presented in Figure 2A,B, respectively, and showed that in the absence of K^+^, aptamers do not form a G4 structure; however, in the presence of increasing amounts of K^+^, a parallel topology is adopted (at least by most conformers in AS1411-N6), as evidenced by positive and negative peaks at 260 nm and 240 nm, respectively. 

In the case of AS1411, increasing K^+^ concentrations above 20 mM did not cause measurable changes in ellipticity, suggesting that aptamer is entirely folded in presence of 20 mM KCl and above (Figure 2B). Conversely, the ellipticity intensities of AS1411-N6 are lower than those of the original oligonucleotide, and are potassium-dependent on the entire concentration range, suggesting that it is not fully folded into a G4 in this range (the plateau is not reached; Figure 2C,D), comparing to AS1411.

Additionally, the CD spectra of AS1411-N6 in the absence of K^+^ present a hyperchromic band around 280 nm compatible with the typical signature of Watson-Crick paired [14,15]. This is consistent with the fact that duplexes can form in absence of potassium, and with the absence of this signal for AS1411. The slight increase of intensity of this band suggests that the G4 formation may somewhat promote the duplex folding. Alternatively, this could reflect possible contributions of hybrid or antiparallel conformers. At the very least, the formation of G4 does not seem to disrupt the duplex.

Given that the CD spectrum revealed a major contribution of the parallel topology, the 5′ end and 3′ flanking sequences should not be on the same face of G4, and therefore the duplex cannot be formed over a quartet. Rather, we hypothesize that the duplex can form (i) intra- or intermolecularly when the G4 is not formed (this is supported by the band at 280 nm in absence of potassium), (ii) intramolecularly, orthogonally to the folded G4 [16], and (iii) intermolecularly between two G4 units. The presence of intermolecular species will be discussed at a later point in this manuscript.

The thermal stability of AS1411-N6 and AS1411 was investigated by CD-melting experiments, in the absence and presence of increasing concentrations of K^+^ monitored at 260 nm, at a 10 μM strand concentration (Figure S1).

Overall, this data evidenced that AS1411 has higher thermal stability comparing to AS1411-N6 (~15 °C in the 40–140 mM KCl range). The results are summarized in Table S1. The duplex formation on AS1411-N6 can have a locking effect on the polymorphic nature of the AS1411 G4 structure (this will be detailed discussed below in the NMR experiment). Taking this data into attention can be concluded that the duplex insertion results in decreased thermal stability, probably due to the withdrawing degrees of freedom from the oligonucleotide to adapt to temperature variation. Differently, these freedom degrees are maintained in AS1411, which, helped by its polymorphic character [10], favors a better adaptation and a transition between different conformations allowing greater resistance to temperature variation.

**Figure 3:**
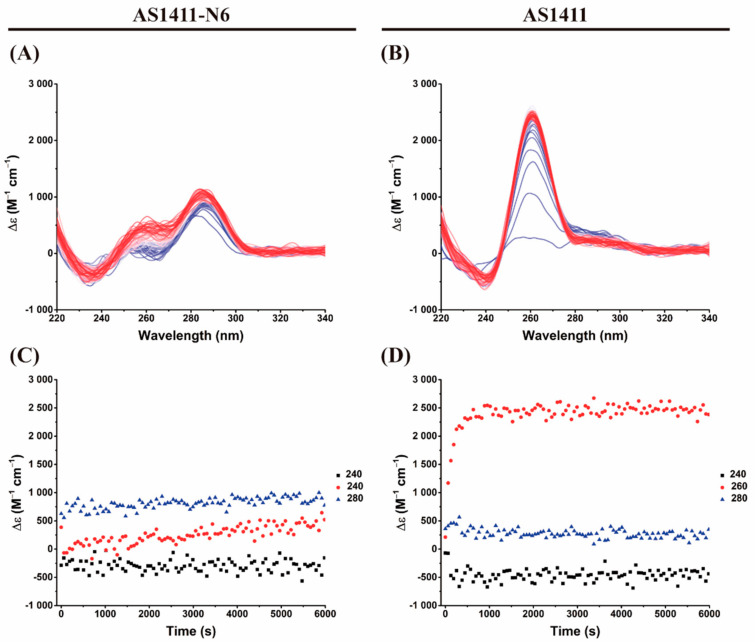
Time-dependent CD experiments of (**A**) AS1411-N6 and (**B**) AS1411 after the addition of 20 mM of KCl. Plots of the time-dependence of three characteristic bands (**C**) AS1411-N6 and (**D**) AS1411. The transition from blue to red represents the time evolution of the CD signal for characteristic bands.

To obtain the kinetic parameters of the G4 formation, we performed a time-dependent CD experiment. The registered features in the time-dependent CD spectrum are also consistent with the K^+^ titrations and are presented in Figure 3. For AS1411, it was verified that the transitions from an unfolded state to a folded parallel G4 was completed in about 10 min (k = 0.0066 s^−1^ from the 260 nm data; Figure 3B,D). Similarly, for AS1411-N6, which initially has features of a duplex structure, the band at 260 nm increases with time, indicating the folding into a G4 (Figure 3A). The 280 nm band is retained (and even increases slightly with G4 formation), in accordance with the K^+^ titration experiments. Also, it is confirmed that AS1411-N6 is far from being fully folded into a G4 in these salt conditions, unlike AS1411, as already evidenced in titration and melting experiments. Consequently, the calculation of kinetic parameters for AS1411-N6 in these environmental conditions (10 mM LiCaCo + 20 mM KCl) was not possible: the reaction is very slow compared to the time interval that is explored; the points are almost linear as shown in Figure 3C. This can be partly explained by the lower stability of modified aptamer and/or in part by the intermolecular nature of some of the conformers.

**Figure 4:**
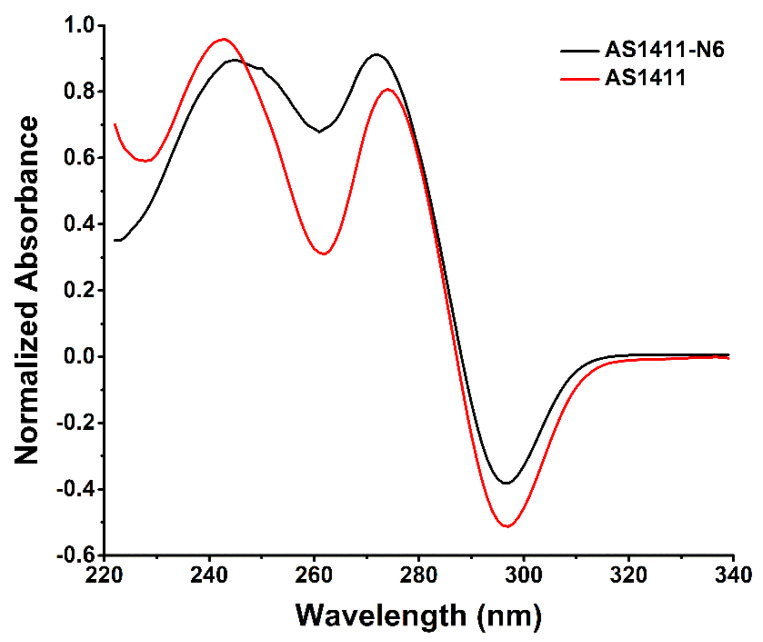
TDS spectra of AS1411-N6 and AS1411, performed in 10 mM of lithium cacodylate supplemented with 140 mM KCl. The graph was obtained by subtracting the spectrum at 25 °C from the spectrum at 90 °C, and then normalized relative to the maximum absorbance.

To complement the CD spectroscopy, TDS analysis was applied to monitor the difference between the unfolded spectrum (high temperature) and the folded spectrum (low temperature) above and below *T*_m_ values (Figure S2) [17,18]. Each TDS signature is specific to each nucleic acid secondary structure and can be considered a “fingerprint” to distinguish different G4s [17,18]. The TDS spectra are depicted in Figure 4. The results showed for both oligonucleotides two major positive peaks: at 245 nm and 272 nm for AS1411-N6 and 243 nm and 274 nm to AS1411 aptamer. These values are in agreement with Mergny et al. that postulated characteristic peaks at 243 ± 2 nm and 273 ± 1 nm for G-rich sequences [17]. Thus, it can be inferred that both oligonucleotides adopt a G4 configuration, confirming the CD data previously presented. Additionally, it is registered a common negative peak at 295 nm for both oligomers. Peak intensity is more accentuated for the AS1411. This peak could be explained by hypochromism due to n→π* and π→π* transitions of each base and resultant of the tetrads stacking in the folding [17]. This peak wavelength is typical of G4 topology according to the literature [18,19].

The TDS factors for both oligonucleotides are presented in Table 1 and were determined according to Karsisiotis et al. (listed in Table 2, Materials and Methods) [18].

Firstly, and considering the TDS ratios for AS1411 (ΔA_240_/ΔA_295_ = 1.89, ΔA_255_/ΔA_295_ = 1.07 and ΔA_275_/ΔA_295_ = 1.62), all denote the presence of an antiparallel topology. However, this result is different from that was indicated and obtained in CD spectroscopy, suggesting the polymorphic nature of AS1411 and its capacity to form antiparallel dimers [10].

AS1411-N6 present a mixture of G4 topologies, parallel and antiparallel, as evidenced by factor values (ΔA_240_/ΔA_295_ = 2.27, ΔA_255_/ΔA_295_ = 2.12 and ΔA_275_/ΔA_295_ = 2.34) (Table 2). This evidence could be explained due to the duplex portion in the AS1411-N6 terminus.

Moreover, and for both oligomers, these results show that the TDS technique has limitations for the study of G4, namely, in complex oligonucleotides (with high polymorphism) or with a mixture of secondary structures (duplex and quadruplex portions, as proposed by AS1411-N6). Thus, and in order to overcome this challenge, it is important to study and validate these challenging structures in order to prove if these ratios values for the generality of G4 structures and not for the best known or a restricted group.

**Figure 5:**
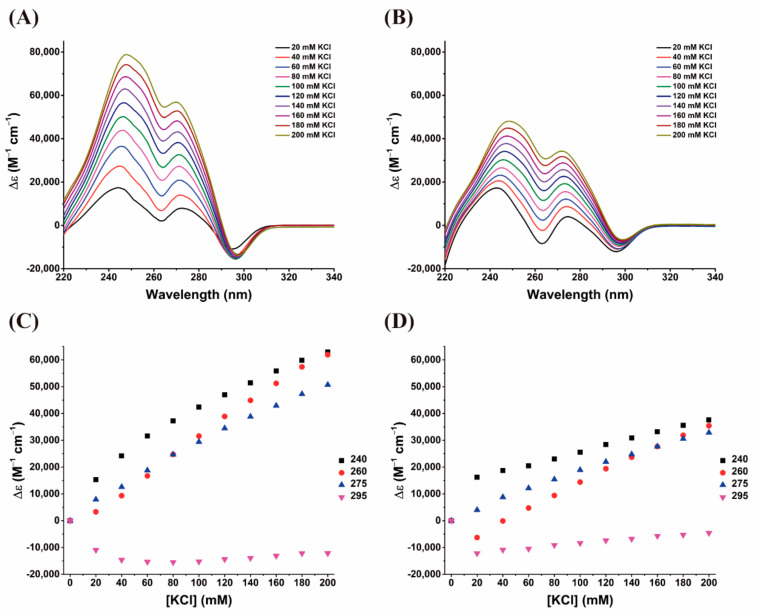
IDS steady-state measurements upon addition of increasing amounts of K^+^. In (**A**,**B**) are depicted the IDS resultant from salt titration performed to both oligonucleotides and in (**C**,**D**) are plotted the absorbance variation in selected wavelengths (240, 260, 275, 295 nm) for AS1411-N6 and AS1411, respectively.

IDS analysis was also performed to elucidate the G4 formation upon the addition of K^+^. IDS is considered a reliable experimental technique to provide specific signatures of different DNA structural conformations [20]. IDS are different from TDS because the absorbance spectra of the unfolded and folded species are temperature-dependent [21], do not involve temperature variation (isothermal process), and reproduce better the absorbance properties of the folding state. IDS is calculated by the subtraction of the UV spectra (Figure S3) acquired in the presence of increasing amounts of K^+^ (folded state) to the spectrum obtained in the absence of K^+^ (unfolded). The IDS experiments for both oligonucleotides are depicted in Figure 5.

The shape of the IDS spectra for both oligonucleotides suggests the formation of a G4 structure, as evidenced by the negative bands at 295 and 260 nm, and the positive bands at 275 and 240 nm, respectively, and the potassium-dependence of their intensity. These values are consistent with those of other G4 oligomers [22,23,24]. Unfortunately, the acquired IDS spectra do not allow to attest the duplex formation in AS1411-N6, as observed in CD experiments by the characteristic CD band.

**Figure 6:**
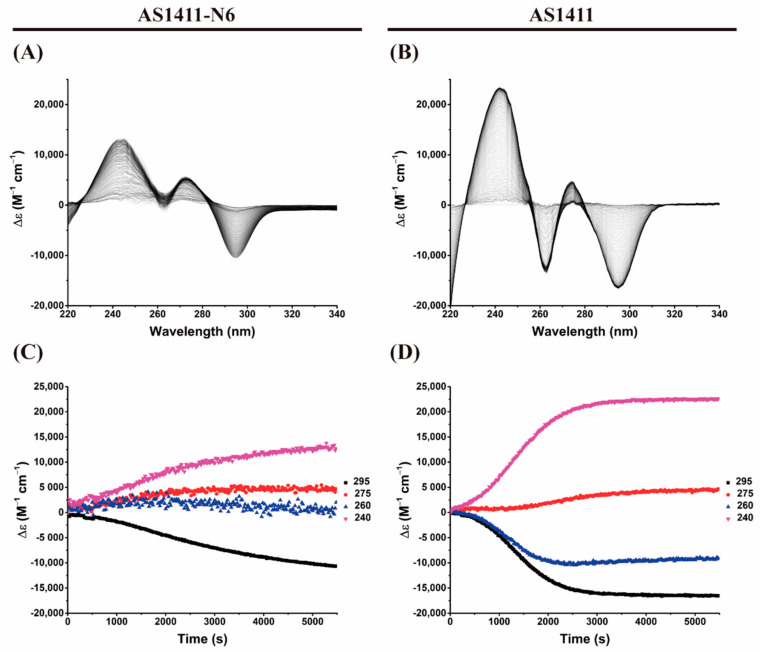
IDS time-dependent spectra for (**A**) AS1411-N6 and (**B**) AS1411 after the addition of 20 mM KCl. The bottom panel represents the time-evolution of the absorbance at selected wavelengths to allow the calculation of kinetic parameters. The kinetic plots (**C**,**D**) correspond to AS1411-N6 and AS1411, respectively.

Similar to CD spectroscopy, IDS time-dependent was employed to determine the kinetic parameters of the G4 formation using 20 mM of KCl to trigger the reaction. Figure 6 depicts the obtained spectra and the plots of the evolution of the characteristic bands.

Time-dependent IDS shows clear transitions from unfolded to folded species. The spectra globally retain the same features as in steady-state experiments, i.e., negative bands at 295 and 260 nm and positive bands at 275 and 240 nm (Figure 6A,B). These results are also similar to previous experiments (steady-state IDS). Only AS1411 is fully folded at 20 mM of KCl, unlike AS1411-N6 (also verified in CD experiments). Considering the measured IDS absorbance at 295 nm, it is possible to draw kinetics plots (Figure 6C,D) and consequently estimate the conversion reaction rates from unfolding to fold states. The calculated kinetic constant to AS1411-N6 is 2.2 × 10^−4^ s^−1^, which is 30 times slower than the AS1411 (1.3 × 10^−3^ s^−1^).

**Figure 7:**
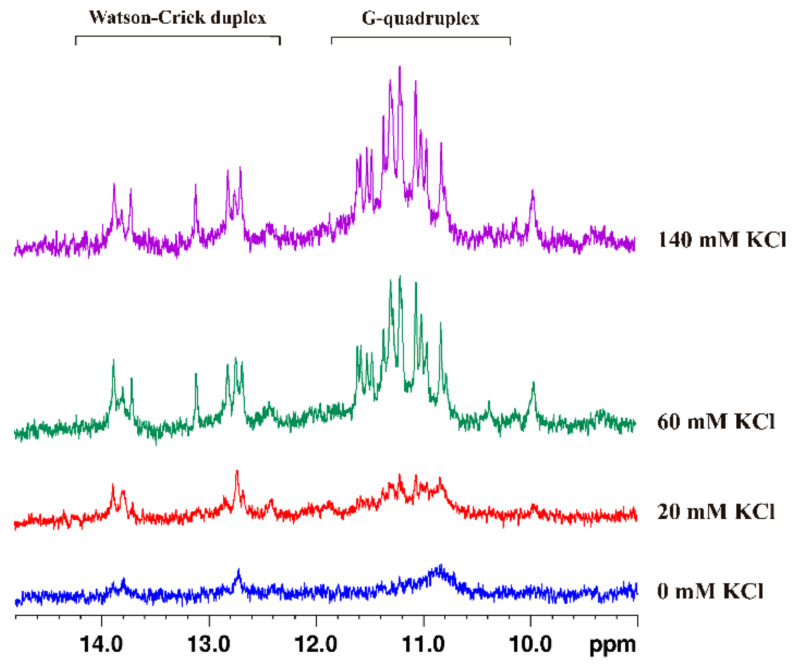
Study of KCl effect in the secondary structure of AS1411-N6 by ^1^H NMR spectroscopy. Two distinct sets of signals are observable upon KCl titration, corresponding to duplex base pairing and G4 portion. Shown on top of the image are these signal sets, detached.

Still to assess the effect of the cation on the secondary structure of AS1411-N6 ^1^H NMR spectroscopy was employed. To this end, a KCl titration was performed directly in the tube with increasing concentrations of salt. The ^1^H NMR titration spectra are displayed in Figure 7.

It is observed that, upon titration with KCl, two distinct sets of signals were visible. The first set corresponds to the guanine imino protons, between 10.5 and 12 ppm, typical of G4 secondary structures. The other set is located at a low magnetic field, between 12.5 and 14 ppm, and corresponds to the Watson-Crick duplex extension added to AS1411-N6. Thus, it can be concluded that both types of structures occur concomitantly in solution, and the increase in ionic strength stabilizes the oligonucleotide. Remarkably, AS1411-N6 shows a well-defined set of the imino protons, being indicative of a single dominant G4 conformation, unlike the precursor AS1411 that is widely described as structurally polymorphic [10,11]. It can be hypothesized that the added Watson-Crick duplex portion can have a locking effect on the natural polymorphism of AS1411. This can open a new pathway for the first detailed structural characterization of AS1411 and, in the future, be seen as a strategy for the structural determination of highly polymorphic oligonucleotides. The sequence in study also stands out for the fact of not having any mutation in the primary original aptamer sequence, unlike the AT11 and their variants, when added or swapped by G-to-T [11,25].

Additionally, to access the effect of temperature on the double secondary structure of AS1411-N6, the temperature was gradually increased in the ionic conditions of the previous titration. The ^1^H NMR spectra are depicted in Figure S4. Firstly, it is demonstrated that an increase in temperature leads to an intensity decrease and change in the shape of signals, and also, to a continuous deviation from initial signal positions. Considering the spectra at 45 °C temperature, it is shown that the duplex signal is the first to vanish, keeping only the G4 portion. This phenomenon is likely due to the lower thermal stability of the duplex compared to the G4 region. Already at 55 °C, the G4 signals look to disappear and are consistent with the T_m_ previously obtained in the CD melting experiment. At 60 °C, it is just residuals that are visible.

After studying the G4-forming AS1411-N6 and the duplex formation in the presence of K^+^ in the first part of this work, which provides insights into sequence and cation dependent folding to NCL recognition, we studied in the second part the binding properties of ligands TMPyP4, PhenDC3, PDS, 360A, and BRACO-19 to AS1411-N6 (Figure 1B).

These studies allow us to evaluate the effect of ligands in terms of G4 stability, binding, topology maintenance, and if the formation of complexes (ligand/AS1411-N6 and ligand/AS1411) influence the recognition of NCL.

**Figure 8:**
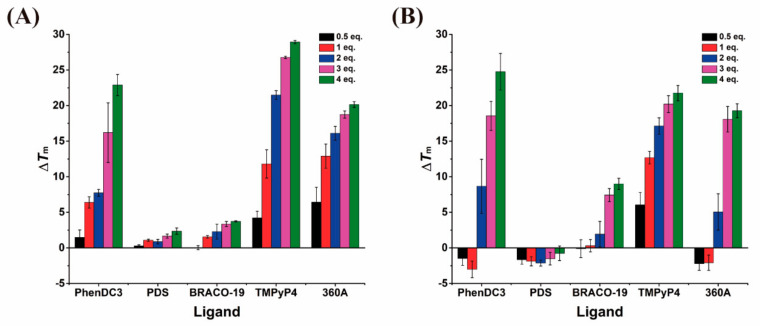
Comparison between thermal stabilization of (**A**) AS1411-N6 and (**B**) AS1411 induced by ligands obtained by FRET-melting experiments. The commercial ligands belong to distinct chemical families as acridines (BRACO-19), pyridines (PDS and 360A); phenanthrolines (PhenDC3), and porphyrin (TMPyP4).

FRET-melting experiments were first performed to measure the stabilization and selectivity of G4 ligands towards AS1411-N6 and AS1411 at several concentrations of ligand. Figure 8 illustrates the induced Δ*T*_m_ (°C) in G4 oligomers by the ligands. Temperature variation was calculated by subtracting the *T*_m_ in the absence and presence of ligand.

These results (Figure 8 and Table S2) evidence that the ligands stabilize G4s in a concentration-dependent manner. Higher Δ*T*_m_ values were obtained for higher concentrations of ligands. The ligand-induced stabilization trend for AS1411-N6 is (at 4 eq.): TMPyP4 > PhenDC3 > 360A > BRACO-19 > PDS; while for AS1411 the trend is PhenDC3 > TMPyP4 > 360A > BRACO-19 > PDS. The higher stabilization of AS1411-N6 by TMPyP4 can be explained by its ability to bind indiscriminately to both G4 and Watson–Crick duplex [26,27], rather than to the G4 only. For comparison, TMPyP4 gave values lower by ~7.3 °C for AS1411 at 4 eq., which has no duplex portion. PDS did not induce stabilization to the oligonucleotides, contrary to 360A, despite both ligands having a pyridine motif in the chemical structure. The difference could be explained due to the presence of the methoxyethan-1-amine groups in PDS, which increases the ligand size and can influence the interaction with the tetrads, affecting the stabilization. The ligand PhenDC3 gave identical ∆*T*_m_ values of 23 to 25 °C for AS1411-N6 and AS1411, respectively. This small difference in stabilization can be explained by the ligand’s ability to selectively bind to G4 structures. The BRACO-19 ligand reveals a lower stabilization effect, comparable to PDS, in AS1411-N6. However, in AS1411, the acridine ligand does not have the same behavior, and shows more thermal stabilization, even comparing to PDS.

Thus, the FRET melting screening enabled the selection of PhenDC3, 360A, and TMPyP4 for the following biophysical assays, based on their AS1411-N6 stabilization ability.

**Figure 9:**
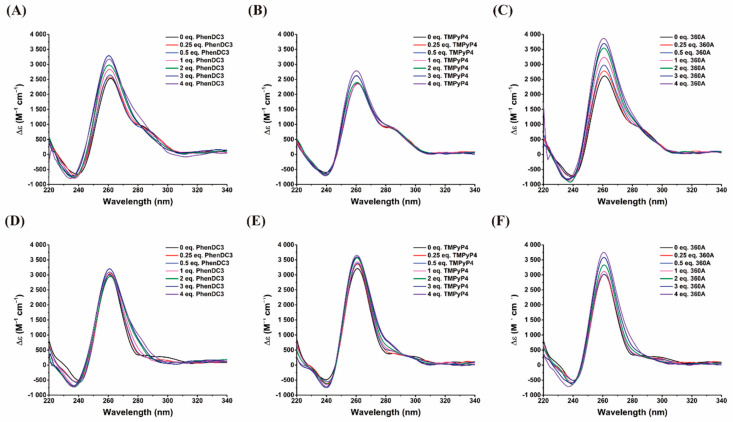
CD spectra of AS1411-N6 (**A**–**C**) and AS1411 (**D**–**F**) in the absence and presence of PhenDC3, 360A, and TMPyP4. Spectra were acquired in 10 mM lithium cacodylate buffer containing 140 mM KCl.

CD spectroscopy was performed to characterize the complexes formed by these selected ligands, AS1411-N6 and AS1411. The ligand effects on the structural conformation of both oligonucleotides were evaluated and presented in Figure 9.

In general, and for both oligomers, an increase was observed in ellipticity and retention of the G4 characteristic bands (not occur the conversion among G4 topologies) after ligand addition. These are indicative of interaction and complex formation between ligand and G4s.

360A ligand promotes the highest ellipticity for both oligonucleotides, followed by PhenDC3 and TMPyP4 to AS1411-N6 and TMPyP4 and PhenDC3 to AS1411, respectively. As described previously, TMPyP4 has the ability to bind to both G4 and duplex [26], explaining the minor increase in ellipticity at 260 nm. The CD spectra of AS1411-N6 at 3–4 molar eq. of PhenDC3 and 360A shows the disappearance of the band at 280 nm (Figure 9), suggesting the disruption of the upon complex formation. Concomitantly, PhenDC3 seems to promote the complete folding of AS1411-N6 in a G4, as shown by comparing the two oligonucleotides at the same ligand concentration (Figure S5). As reported in literature these ligands can induce transitions between G4 conformations [28,29]; however, none show the capacity to disrupt another secondary structure to induce the G4 formation.

Thermal stabilization of AS1411-N6 and AS1411 in the presence of PhenDC3, 360A, and TMPyP4 was studied using the CD melting experiment. Thermal melting of AS1411-N6 and AS1411 was monitored at 260 nm. The *T*_m_ and melting curves are presented in Table S3 and Figure S6, respectively. The *T*_m_ value was observed around 55 and 69 °C for AS1411-N6 and AS1411 without ligand, respectively. 

We observed that the interaction of 360A and PhenDC3 (at 4 eq.) with AS1411-N6 enhanced the thermal stability by more than 30 °C for both oligonucleotides. This confirms that a complete folding of the G4 is achieved at room temperature upon binding of the PhenDC3 and 360A ligands. Both ligands stabilize AS1411 to a lower extent. However, it is not advised to carry out direct comparisons of Δ*T*_m_ for oligonucleotides that have significantly different *T*_m_ (this is the case here) [30]. In fact, at high ligand stoichiometries (3 or 4 eq.), the melting temperatures of the complexes are lowered (Phen-DC3) or increased (360A) in the presence of the flanking sequences to a non-statistically significant extent. Therefore, in absence of duplex in AS1411-N6, the flanking sequences do not significantly alter ligand binding.

The non-selective TMPyP4 yielded a lower thermal stabilization than its G4-selective counterparts (~23 and ~25 °C for AS1411-N6 and AS1411, respectively). Note that, given that TMPyP4 could bind to both the G4 and duplex region, and that the melting temperature was derived from the signal at 260 nm (i.e., reflecting the G4 melting), it is possible that the stability of the AS1411-N6 complex is underestimated.

The Δ*T*_m_ acquired by FRET-melting are lower but close to those obtained by CD-melting. This is likely due to the fact that, here, the FRET efficiency is largely dependent on the duplex melting (given that the fluorophores are linked to the flanking sequences), which is less stable than the G4 region (as seen in the temperature-dependent NMR experiments). Conversely, the CD-melting is more specifically following the G4 melting.

Then SPR biosensor was employed to quantify the binding affinity between AS1411-N6 and AS1411 G4s with PhenDC3, 360A, and TMPyP4. Streptavidin sensor chips were used to capture biotin-labeled AS1411-N6 and AS1411. The binding curves are presented in Figure S7 and displayed a complex behavior characterized by a fast association phase and a slow dissociation, which made it necessary to include a glycine pH 2.5 regeneration step. Affinity constants indicated that all the ligands bound G4s with high affinity (Table 3), in particular 360A. These affinities are in line with the large stabilizations observed in melting experiments.

**Figure 10:**
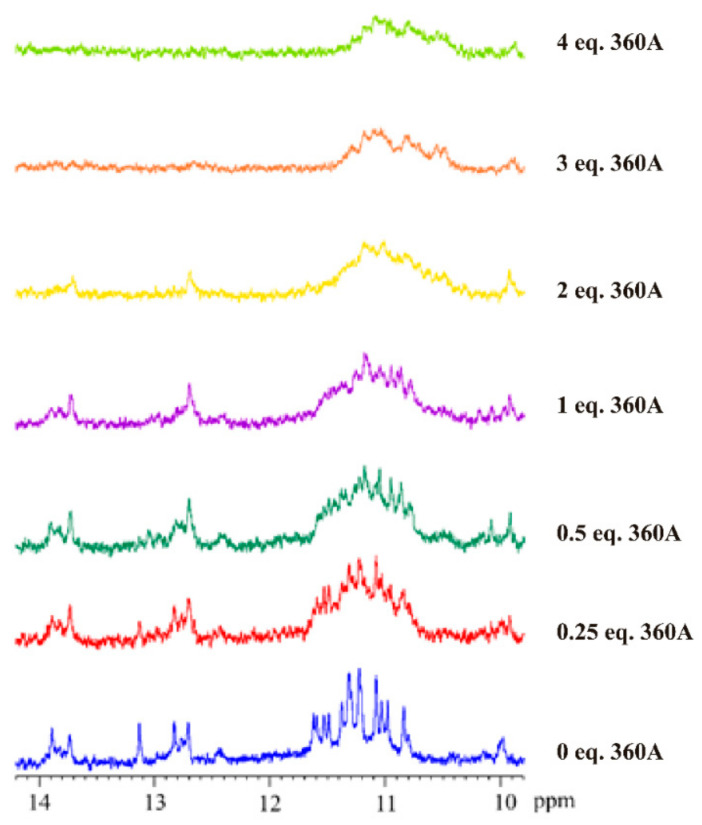
^1^H NMR spectra showing the imino region of AS1411-N6 upon 360A titration. The spectra were recorded in 90% H_2_O, 10% D_2_O at 25 °C.

The complex formation of AS1411-N6 and 360A was followed by ^1^H NMR spectroscopy. 360A was chosen because it showed a significant stabilizer effect (>30 °C) and has the highest affinity (*K*_D_ = 10^−8^ M). 360A is a well-known strong G4 ligand with negligible interaction with other DNA conformations [27,31]. The ^1^H NMR spectra of the AS1411-N6 upon 360A titration are shown in Figure 10.

A set of sharp peaks in the AS1411-N6 G4 spectrum is visible in the imino proton region (10–12 ppm). Upon titration with 360A, the NMR spectra showed a pronounced effect of the G4 ligand on the imino pattern of AS1411-N6, since their relative peak intensities diminished, and the broadening is remarkable. The broadened and poorly resolved spectra, might be caused by dimerization, and/or indicate less-defined binding of 360A to AS1411-N6 [32]. Furthermore, after the addition of 3 molar equivalents, the spectrum suggests a reorganization of the G4 structure, once the peaks related to duplex conformation (12–14 ppm) are not detectable. Therefore, the binding of 360A to AS1411-N6 suggests a different folding pattern of the G4 structure. The NMR profile is in agreement with CD experiments, which suggests the disruption of the duplex conformation upon titration with 2 eq. 360A.

Overall, the ligands bind and stabilize the G4 in AS1411-N6. The NMR spectra show a well-resolved spectrum, indicative of a dominant G4 conformation. However, the ligand-induced G4 AS1411-N6 polymorphism needs to be determined in future work as done for AS1411 [10].

We further assessed the binding of AS1411-N6 to NCL with and without ligands by fluorimetric titrations. AS1411-N6 labeled with 5′-FAM and 3′-DABCYL was used to record fluorescence emission spectra. As seen in Figure S8, upon excitation at 495 nm, 5′-FAM emitted fluorescence as a broad band centered at 518 nm. After the addition of NCL RBD 1,2 solution, an enhancement was verified in the fluorescence intensity, resultant of the break of FRET phenomena due to the increase in fluorophores distance provoked by the interaction of AS1411-N6 with NCL RBD 1,2. In this experiment, a ≈3-fold increase in fluorescence units was observed, denoting a strong interaction between the NCL RBD 1,2 and AS1411-N6 (Figure S8).

*K*_D_ values were calculated through variation in the fluorescence intensities in the absence and presence of increasing amounts of NCL RBD1,2 as described previously [33]. The fluorescence intensity values were then fitted to a saturation binding model and the *K*_D_ values, summarized in Table 4, were determined using non-linear regression analysis (Equation (7); Figure S8). The binding affinity among protein and modified aptamer in the micromolar range are indicative of high affinity and stable complex formation between the NCL RBD 1,2 and AS1411-N6, as seen in AS1411 [34]. Additionally, for AS1411-N6, the influence of ligands in the recognition of NCL RBD 1,2 was tested. The fluorescence spectra (Figure S8) showed for all ligands a ≈2-fold increase in fluorescence units, denoting their influence on recognition and interaction of NCL RBD 1,2 when compared with ligand-free AS1411-N6.

This conclusion is supported by the *K*_D_ values for AS1411-N6/ligand complex and protein (Table 4). The results evidence that complexation with 1 eq. of 360A increase the binding affinity of AS1411-N6 to NCL. On the other hand, the complex formation with TMPyP4 and PhenDC3 decreased the binding affinity of AS1411-N6 to NCL. Indeed, PhenDC3 was previously described to disrupt and prevent the binding of G4 structures to NCL [35].

**Figure 11:**
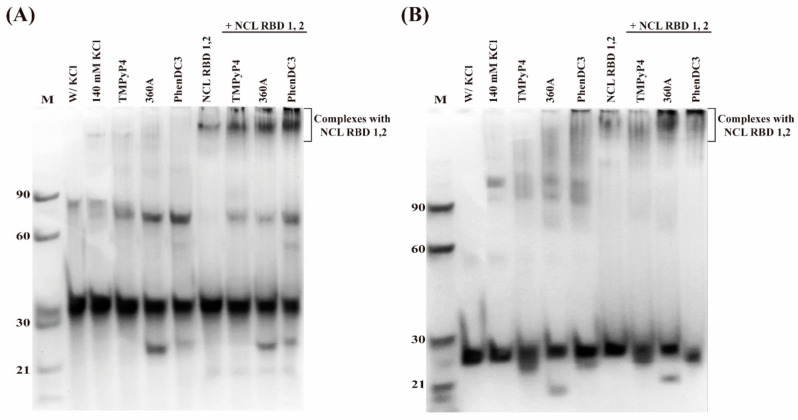
Polyacrylamide gel electrophoresis under native conditions of (**A**) AS1411-N6 and (**B**) AS1411. The gel was supplemented by 140 mM KCl. Migration markers were oligothymidylate single-stranded DNA (n = 21, 30, 60 and 90). Lane 1—migration marker; lanes 2 and 3—aptamer in the absence and presence of KCl; lanes 4 to 6—aptamer/ligand complexes (with 1 molar eq. of TMPyP4, 360A and PhenDC3, respectively); lane 7—aptamer with NCL; lanes 8 to 10—aptamer/ligand complexes (at same order) with NCL.

The formation of the complexes AS1411-N6/NCL RBD 1,2 and AS1411/NCL RBD 1,2 in the absence and presence of PhenDC3, 360A, and TMPyP4 was also evaluated by PAGE. The oligonucleotides were diluted to 20 μM and complexes with ligands and/or NCL RBD 1,2 were prepared at a 1:1 molar ratio. The results are presented in Figure 11. As expected, despite the general similarity in electrophoretic mobilities of AS1411-N6 and AS1411, there are subtle differences that could be of utmost importance to understand the structural polymorphism of both oligos. In the presence of 140 mM KCl, the two oligomers migrate mostly as a single major intense band, attributed to the unimolecular monomeric form. However, other retarded bands are observed, suggesting multiple conformations in solution. In the particular case of AS1411-N6, the retarded band runs below the 90 nt DNA marker band, suggesting the presence of a dimeric form, which has a molecular size of 76 nucleotides. On the other hand, the retarded band observed for AS1411 runs above the 90 nt DNA marker band, which is in agreement with the intermolecular tetramer parallel G4 comprising 104 nucleotides [10].

In the presence of G4 ligands, the electrophoretic profile of AS1411-N6 highlighted an increase in the band intensity of the dimeric form and a slight increase in mobility when compared with aptamer in the absence of ligand. This could denote a different dimer because the initial dimer could be templated by an intermolecular duplex, but not in the presence of the ligand. In contrast, the bands corresponding to the tetrameric G4 of AS1411 are diffused, as shown by the polyacrylamide gel. 

The binding between AS1411-N6 and NCL RBD 1,2 was also assessed and compared with AS1411 in absence of ligands. Upon addition of NCL RBD 1,2, it is worth noting that the bands corresponding to the dimeric and tetrameric forms were not visible in the electrophoretic profile, suggesting the binding of those molecular forms to NCL RBD 1,2 or also that the equilibrium is displaced towards the monomers, via their binding to NCL. 

The major differences in the electrophoretic profile were observed in the presence of G4 ligands and NCL RBD 1,2. The electrophoretic profile of AS1411-N6 showed bands of the dimeric form in the presence of G4 ligands and RBD 1,2, while in the electrophoretic profile of AS1411, the bands of tetrameric form were absent, indicating mainly the formation of the monomeric form.

## 3. Materials and Methods

### 3.1. Oligonucleotides and Reagents

Solutions were prepared with ultrapure water (18.2 Ω cm^−1^ resistivity), purified with a milli-Q system from Millipore (USA). AS1411 (5′-GGTGGTGGTGGTTGTGGTGGTGGTGG-3′) and AS1411-N6 (5′-GGTTGGGGTGGTGGTGGTTGTGGTGGTGGTGGCCAACC-3′) were purchased lyophilized from StabVida (Lisbon, Portugal) and Eurogentec (Liège, Belgium) with double-HPLC purification and used without further treatment (Figure 1A). The biotinylated and fluorophore-labeled oligonucleotides were acquired from Eurogentec (Liège, Belgium). Using the molar extinction coefficient (ε) provided by the manufacturer and the absorbance at 260 nm with a UV–Vis spectrophotometer (Thermo Scientific™ Evolution 201), the concentrations of oligonucleotides were determined. Oligonucleotides solutions were stored at −20 °C until used and for the experiments were prepared in the buffer, heated at 99 °C for 3 min, and slowly cooling down to room temperature.

The ligands PhenDC3 (3,3′-[1,10-phenanthroline-2,9-diylbis(carbonylimino)]bis[1-methylquinolinium] 1,1,1-trifluoromethanesulfonate, CAS: 929895-45-4), PDS (3-{1-[3-(dimethylamino)propyl]-2-methyl-1H-indol-3yl)-1H-pyrrole-2,5-dione; CAS: 1085412-37-8), BRACO-19 (N,N′-(9-(4-(dimethylamino)phenylamino)acridine-3,6-diyl)bis(3-(pyrrolidin-1-yl)propanamide); CAS: 1177798-88-7), TMPyP4 (tetra-(N-methyl-4-pyridyl)porphyrin; CAS: 36951-72-1) and 360A (2,6-N,N′-methyl-quinolinio-3-yl)-pyridine dicarboxamide); CAS: 794458-56-3) were obtained from Sigma-Aldrich (St. Louis, MO, USA). The chemical structures of each ligand are represented in Figure 1B. The recombinant NCL peptide (RBD 1,2 domains) was purchased from NZYtech (Lisbon, Portugal).

### 3.2. Circular Dichroism (CD) Spectroscopy

CD spectroscopy was performed using a Jasco J-815 CD spectropolarimeter equipped with a Peltier-type temperature controller (CDF-426S/15 model). Spectra were recorded on the 200–340 nm range (scanning speed of 200 nm/min, 1 nm bandwidth, 1 s integration time over 5 averaged accumulations) at 25 °C in 1 mm path-length quartz cuvettes. For KCl titrations, the oligonucleotides were previously diluted in lithium cacodylate buffer (10 mM, pH = 7.2) and the required volume of monovalent cation was added directly to the quartz cell. Spectra were recorded after 30 min of incubation after salt addition. The data were converted to molar dichroic absorption (Δε), through Equation (1), where *θ* is ellipticity in millidegrees, *c* is the oligonucleotide concentration (mol L^−1^), and *l* is the path of the length in cm.

(1) $$\Delta \varepsilon =\;\frac{\theta }{32,980\;\times \;c\;\times \;l}$$

The melting experiments were performed after each titration point. The data were collected at the specific wavelength of maximum ellipticity (260 nm). The temperature gradient ranged from 20 °C to 100 °C with a rate of 4 °C/min and 5 s of equilibration in each point. The ellipticity was normalized into fraction folded (f) plots according to Equation (2) and was fitted to a Boltzmann distribution using OriginPro2016:

(2) $$f=\;\frac{CD-CD_{\lambda }^{min}}{CD_{\lambda }^{max}-\;CD_{\lambda }^{min}}$$

where CD is the ellipticity at each temperature and CD_min_ and CD_max_ is the lowest and highest ellipticities, respectively, allowing the determination of melting temperatures (*T*_m_). Additionally, also performed was a titration of commercial ligands following all parameters and incubation times described above.

In time-dependent experiments, aptamers were also diluted in lithium cacodylate buffer, and to trigger the G4 formation, 20 mM of KCl was added directly in the cuvette. Spectra were also acquired at 25 °C between 200 and 340nm (scan speed of 200 nm/min, 1 nm bandwidth, 1s integration time) in 1 mm path-length quartz cuvettes. The characteristic wavelengths (240, 260, and 295 nm) were fitted with a single (*j* = 1) and double-exponential (*j* = 2) model, following the Equation (3), where Δε_0_ is the offset, A_i_ is the span constant and *k_i_* is the rate constant of the process *i*.

(3) $$\Delta \varepsilon ={\Delta \varepsilon }_{0}+\;\overset{j}{\underset{i=1}{\sum }}\left[A_{i}\exp\left(-k_{i}t\right)\right]$$

According to [21], the quality of models was evaluated applying the extra sum-of-squares F tests and the corrected Akaike information criterion. Further, the half-life (t_1/2)_, and time constant τ were calculated following their classical definition presented in Equations (4) and (5).

(4) $$t_{\frac{1}{2}}^{i}=\;\frac{\ln\left(2\right)}{k_{i}}$$

(5) $$\tau_{i}=\;\frac{1}{k_{i}}$$

### 3.3. UV Absorption Spectroscopy

UV experiments were performed using a Thermo Scientific TM Evolution TM201 UV–Vis spectrophotometer (Thermo Fisher Scientific, Waltham, MA, USA) and recorded between 220–340 nm. Spectra acquisition was conducted with a scanning rate of 600 nm/min, 1 nm data intervals, and 0.05 s of integration time. For all experiments, the oligonucleotides were used at 5 μM concentration in lithium cacodylate buffer (10 mM, pH = 7.2).

Thermal difference spectra (TDS) were carried out at 90 °C and 25 °C, which correspond to the unfolded and folded states, respectively. The TDS spectrum was calculated by subtracting the 25 °C spectra from the obtained at 90 °C. The difference spectra were normalized relative to the maximum absorbance. In this experiment, the oligonucleotides samples were supplemented with 140 mM KCl. For TDS analysis, the UV TDS factors described by Karsisiotis et al. [18] were considered, presented in Table 2.

Isothermal difference spectra (IDS) were acquired at 25 °C and calculated by subtraction of the UV spectra of oligonucleotides, acquired in the absence or presence of increasing amounts of KCl. IDS and TDS are not directly comparable due to the fact of the folding state varies according to temperature; however, the isothermal experiment reproduces the absorbance properties of the folding state [21].

Time-dependent IDS were performed by the addition of 20 mM KCl to the 5 μM of oligonucleotides following the next parameters: scan every 20 s between 220−340 nm, scanning rate of 1200 nm/min, 1.0 nm step, and 0.05 s integration time).

### 3.4. Nuclear Magnetic Resonance (NMR) Spectroscopy

Standard zgesgp ^1^H NMR spectrum was recorded on Bruker Avance 600 MHz (Bruker Avance III). The spectrometer was equipped with a QCI cryogenic probe. The AS1411-N6 samples were prepared in potassium phosphate buffer (10 mM, pH 6.9) supplemented with 10% D_2_O. NMR titration was performed by titrating increasing amounts of KCl directly to the 3 mm tube followed by the annealing step as described above.

1D spectrum acquisition. The following experiments are therefore carried out at different temperatures (25, 37, 45, 50, 55, and 60 °C) in order to check the topological conversion. In ligand titration experiments, aliquots of the 360A stock solution were directly added to the oligonucleotide solutions inside the NMR tube. All spectra were acquired and processed with the software Topspin 3.1. Chemical shifts (δ) were measured in ppm.

### 3.5. Fluorescence Resonance Energy Transfer (FRET) Melting

FRET melting experiments were performed using a CFX Connect™ Real-Time PCR Detection System (Bio-Rad, Hercules, CA, USA), equipped with a FAM filter (λ_ex_ = 492 nm; λ_em_ = 516 nm). Oligonucleotides at 0.2 µM were annealed in lithium cacodylate (10 mM, pH 7.2) supplemented with 140 mM KCl before the experiment as described in the above sections. Each experimental condition was tested in duplicate in three separate plates. For each condition, 20 μL of oligonucleotides was aliquoted into each strip, followed by 5 μL of commercial ligands solutions, at five different final concentrations (0.5, 1, 2, 3, and 4 eq.). Then, this was followed by an incubation time of 30 min at room temperature. The thermocycler was parametrized to measure and acquire the FAM emission after each step with a stepwise increase of 1 °C every 1 min, from 25 °C to 95 °C. Through the fluorescence normalized curves, specifically to values when normalized emission is 0.5, the *T*_m_ values were ascertained for each type and concentration of ligand.

### 3.6. Surface Plasmon Resonance (SPR) Biosensor

SPR analysis was conducted on a Biacore T200 (Biacore, GE Healthcare, Uppsala, Sweden) with SA (streptavidin-coated sensor chip) serie S sensor chip (GE Healthcare, Sweden).

The biotin-labeled AS1411-N6 (25 nM dissolved in 10 mM phosphate buffer supplemented with 140 mM KCl) was previously annealed. The sensor chip was equilibrated with running buffer (10 mM phosphate buffer supplemented with 140 mM KCl) at 25 µL/min during 1 h until obtained a stable baseline. Activation buffer (1 M NaCl, 50 mM NaOH) was injected for 3 min for a course of seven times to remove unbound streptavidin from the sensor chip using the manual run command. After that, and to ensure surface stability, two primes with running buffer were performed and the buffer was flowed for 10 min to get baseline stability before immobilization at flow rate 1 µL/min. Manual inject was used to immobilize biotin-labeled AS1411-N6 on flow cell 2 (~100 μL of a 25 nM AS1411-N6) and the injection was stopped after the desired level was reached (~250 RU to minimize mass transport effects). Flow cell 1 was used as a control and was left blank for subtraction.

For kinetic/affinity analysis, each ligand was serially diluted in running buffer from a range of concentrations of 1 nm to 1 µM. All experiments were performed in triplicate at 25 °C. Each ligand was injected from low to high concentrations during 75 s with a flow rate of 50 µL/min, followed by dissociation of 600 s. Surface regeneration was achieved by injecting two pulses of 30 s of 10 mM glycine/HCl pH 2.5, and the next three 60 s injections of running buffer to remove any trace of regeneration solution.

BiaEvaluation Software was used for data analysis and the likelihood of fittings was assessed through the statistical parameters of Chi^2^ and U-value. All sensorgrams were double corrected for non-specific binding and refractive index changes (bulk effect) by subtracting the signals of an equivalent injection across the reference flow cell 1. Dissociation constants were obtained from the 1:1 affinity model of sensorgrams.

### 3.7. Fluorescence Titrations

The fluorescence titrations were performed on a Horiba FluoroMax4 fluorometer (Kyoto, Japan) equipped with a Peltier-type temperature control system defined at 25 °C. Before experiments, AS1411-N6, at 1 µM concentration, was annealed in potassium phosphate buffer (10 mM, pH 6.9) supplemented with 140 mM KCl, in conditions previously described (99 °C for 3 min and slow cooling at room temperature). Reference and samples were scanned using a high-precision quartz suprasil cuvette (light path 10 mm × 4 mm). Taking into account the maximum absorbance value of FAM fluorophore, samples were excited at 495 nm and the fluorescence spectra were acquired between 500 and 700 nm (integration time of 0.5 s, an emission and excitation slit fixed at 3 nm and step size of 1 nm, averaged over 3 scans). AS1411-N6 was titrated with increasing concentrations of NCL (RBD 1,2), and the spectra were acquired after 5 min of equilibration at room temperature. Fluorescence data was converted into a fraction of bound ligand (α) plots using Equation (6), where *I* is the fluorescence intensity at 518 nm at each NCL/AS1411-N6 ratio, and I_free_ and I_bound_ are the fluorescence intensity of the free and fully bound NCL (RBD 1,2), respectively.

(6) $$\alpha =\;\frac{I-I_{\lambda }^{free}}{I_{\lambda }^{bound}-\;I_{\lambda }^{free}}$$

A one-site saturation binding function was applied to fit data points, using Origin Pro 2016, according to the following equation (Equation (7)):

(7) $$\alpha =\;\frac{\left[AS1411-N6\right]}{K_{D}+\left[AS1411-N6\right]}$$

where *K*_D_ is the apparent equilibrium dissociation constant and [AS1411-N6] is the concentration of the AS1411-N6.

### 3.8. Non-Denaturing Polyacrylamide Gel Electrophoresis (PAGE) Analysis

Non-denaturing polyacrylamide gel (15%) supplemented by 140 mM KCl was prepared and loaded in a vertical electrophoretic cell Mini-Protean II (BioRad) with a PowerPac™ power supply (120 V, 4 W, 90 min) at room temperature. Before loading on the gel, the samples were mixed with sucrose (Sigma-Aldrich, St. Louis, MO, USA) until a final concentration of 23%.

The oligonucleotides were prepared at 20 µM in 10 mM phosphate buffer with 140 mM of KCl and were incubated with the ligands and NCL RBD 1,2 at a 1:1:1 ratio (ligand/DNA/NCL RBD 1,2) during 30 min for complex formation. Oligonucleotide markers were purchased from NZYTech (21, 30, 60, and 90 nt) and loaded in parallel on the gel. After electrophoresis, the gels were stained by stains-all solution (Sigma-Aldrich, St. Louis, MO, USA) under continuous and gentle agitation overnight followed by discoloration in water under the light before visualization on GE Typhoon Trio Imager Scanner (GE Healthcare, Chicago, IL, USA).

## 4. Conclusions

As concluding remarks, our results suggest a decrease in the structural polymorphism of the G4-forming aptamer AS1411-N6 compared to the original aptamer. By means of CD and NMR spectroscopy, we evaluated the secondary structure of AS1411-N6 and concluded that it forms a duplex/G4 hybrid structure in the presence of K^+^. Additionally, the time-dependent experiments showed similar trends for both AS1411 and AS1411-N6, despite slower conversion reaction rates from unfolding to the folded state of AS1411-N6. The stabilization effect of G4 ligands on both oligonucleotides was evaluated through CD- and FRET-melting experiments and revealed the promising stabilizer effects of PhenDC3, 360A, and TMPyP4. Thereafter, the affinity of those G4 ligands towards AS1411-N6 and AS1411 was evaluated through SPR measurements and showed that 360A is the most prominent affine ligand for both aptamers. The fluorescence titration experiments demonstrated an approximately 3-fold increase in fluorescence at saturating NCL concentrations, suggesting that could be used for further protein detection experiments. The *K*_D_ value of AS1411-N6 to NCL RBD 1,2 was in the micromolar range, indicating that our modification strategy does not affect the binding interactions at the molecular level. Also, the stabilization of AS1411-N6 with 360A led to a remarkable enhancement of the target affinity towards NCL RBD 1,2, while the stabilization with TMPyP4 and PhenDC3 decreased the binding affinity. The ^1^H NMR spectra of the AS1411-N6 upon 360A titration revealed a poorly resolved imino proton region, which suggests a less-defined binding of the ligand to AS1411-N6 and/or AS1411-N6 dimerization. Lastly, PAGE experiments without ligands showed that after the addition of NCL RBD 1,2, the bands corresponding to the dimeric and tetrameric forms were not visible, suggesting the binding of those molecular forms to protein. In light of this observation, we also hypothesize that the equilibrium could be displaced towards the monomers via them binding to the protein.

Overall, our results and strategy open up a framework for the design and development of new engineered G4-forming aptamer beacons, by locking the structure with 5′ and 3′ end complementary strands for the NCL targeting.

## Figures and Tables

**Table 1:** Topology characterization of AS1411 and AS1411-N6 according to reference TDS values.

	ΔA_240_/ΔA_295_	ΔA_255_/ΔA_295_	ΔA_275_/ΔA_295_	Topology
**AS1411**	1.89	1.07	1.62	Antiparallel
**AS1411-N6**	2.27	2.12	2.34	Mixed

**Table 2:** Reference TDS factor values according to the type of topology [18].

ΔA_240_/ΔA_295_	ΔA_255_/ΔA_295_	ΔA_275_/ΔA_295_	Topology
>4	>3.5	>3	Parallel
<2	<1.5	≤2	Antiparallel

**Table 3:** *K*_D_ constant values of AS1411-N6 and AS1411 measured by SPR biosensor.

Oligomer	*K*_D_ (M)		
	PhenDC3	360A	TMPyP4
**AS1411-N6**	2.27 × 10^−7^ ± 5.4 × 10^−8^	3.82 × 10^−8^ ± 5.4 × 10^−9^	2.64 × 10^−7^ ± 3.00 × 10^−8^
**AS1411**	3.44 × 10^−7^ ± 5.38 × 10^−8^	4.34 × 10^−8^ ± 8.47 × 10^−9^	1.15 × 10^−7^ ± 7.09 × 10^−9^

**Table 4:** *K*_D_ constant values of AS1411-N6 and AS1411-N6/ligand complex upon titrated with NCL RBD 1,2.

*K*_D_ (μM)			
AS1411-N6	AS1411-N6/Ligand Complex		
	360A	TMPyP4	PhenDC3
1.0 ± 0.1	0.7 ± 0.1	1.1 ± 0.2	1.5 ± 0.3

## Data Availability

The data presented in this study are available on request from the corresponding author.

## Supplementary Materials

The following are available online at https://www.mdpi.com/1424-8247/14/2/121/s1, Figure S1: CD melting spectra of both oligonucleotides in presence of increasing amounts of KCl. Figure S2: UV spectra of AS1411-N6 and AS1411 in presence of 10 mM of LiCaCo buffer supplemented with 140 mM of KCl, at 25 °C and 95 °C. Figure S3: UV spectra of AS1411-N6 (A) and AS1411 (B) in with increasing amounts of KCl. Figure S4: Study of temperature effect in AS1411-N6 G4 structure by 1H NMR spectroscopy. Figure S5: Comparison of the profile of both sequences due to the effect of high concentrations of ligands, namely, PhenDC3 and 360A at 4 molar equivalents. Figure S6: CD-melting curves of aptamers in presence of several ligands at several concentrations. Figure S7: Equilibrium binding curves of AS1411-N6 and AS1411 upon addition of ligands. Figure S8: Fluorescence spectra and saturation binding plot analysis of AS1411-N6 and AS1411-N6/ligand complex upon NCL RBD 1,2 titration. Table S1: Tm of both oligonucleotides in presence of increasing amounts of K+ measured by CD melting. Table S2: ΔTm of oligonucleotides in presence of commercial ligands by FRET melting assay. Table S3: Tm of AS1411-N6 and AS1411 in presence of PhenDC3, 360A, and TMPyP4 by CD melting experiment.
