# Supplementary material for: Locking up the AS1411 Aptamer with a Flanking Duplex: Towards an Improved Nucleolin-Targeting
Source: Pharmaceuticals (Basel). 2021 Feb 4;14(2):121. doi: 10.3390/ph14020121 (PMC7916057; doi:10.3390/ph14020121)
Supplement: Supplementary file 1 [file pharmaceuticals-14-00121-s001.pdf]

Article

Locking up the AS1411 Aptamer with a Flanking Duplex: Towards an Improved Nucleolin-Targeting

André Miranda [1], Tiago Santos [1], Eric Largy [2] and Carla Cruz [1,*]

[1] CICS-UBI—Centro de Investigação em Ciências da Saúde, Universidade da Beira Interior, Av. Infante D. Henrique, 6200-506 Covilhã, Portugal; andre.miranda@ubi.pt (A.M.); tiagoaasantos@hotmail.com (T.S.)
[2] Université de Bordeaux, INSERM & CNRS, Laboratoire Acides Nucléiques: Régulations Naturelle et Artificielle, (ARNA, U1212, UMR5320), IECB, 2 rue Robert Escarpit, 33607 Pessac, France; eric.largy@u-bordeaux.fr
* Correspondence: carlacruz@fcsaude.ubi.pt

Supplementary information

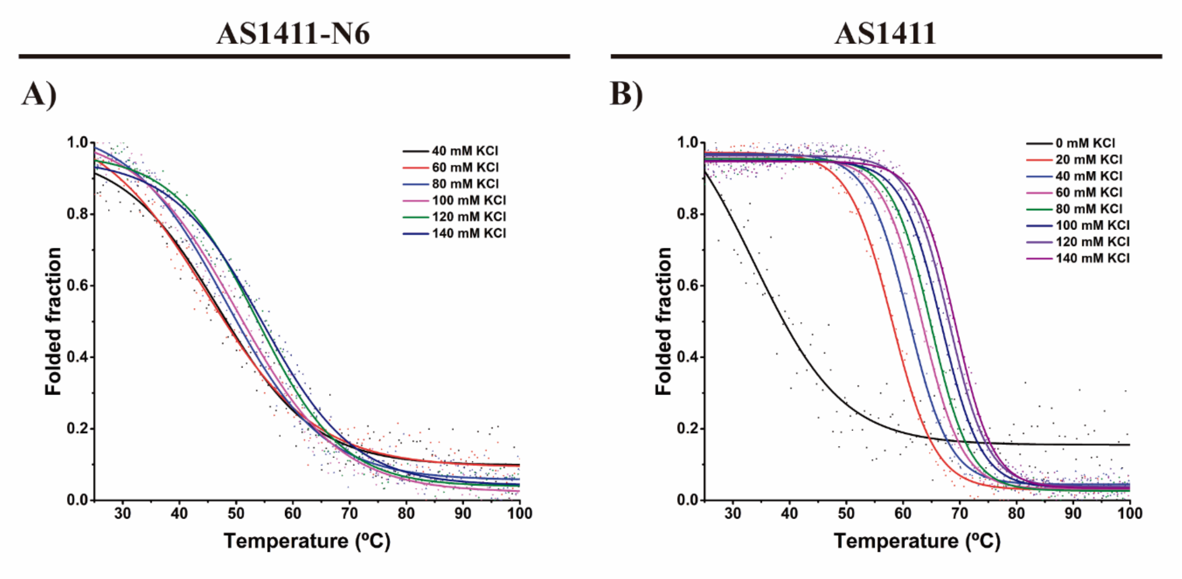

Figure S1. CD melting spectra of both oligonucleotides in presence of increasing amounts of KCl. Ellipticity variation was measured following the wavelength at maximum ellipticity. To AS1411-N6 (A) was not observed two-state transition to 0 and 20 mM of KCl.

Table S1. T_m of both oligonucleotides in presence of increasing amounts of K^+ measured by CD melting.

**[K^+] (mM)**	***T_m* (ºC)**	
	**AS1411-N6**	**AS1411**
**0**	- *	33.7 ± 2.9
**20**	- *	58.1 ± 0.1
**40**	44.3 ± 0.9	60.9 ± 0.1
**60**	46.9 ± 0.9	63.4 ± 0.1
**80**	48.3 ± 0.5	64.9 ± 0.1
**100**	50.3 ± 0.4	66.7 ± 0.1
**120**	53.7 ± 0.3	68.1 ± 0.1
**140**	56.6 ± 0.3	69.2 ± 0.1

***** No two-state transition was observed for CD melting experiments

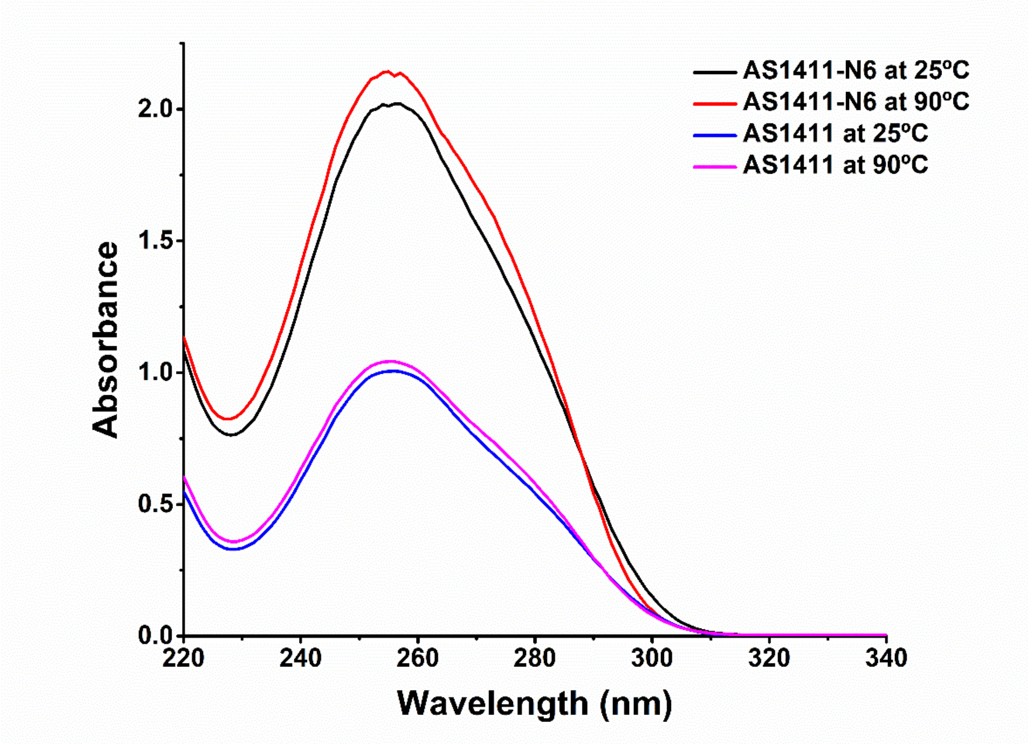

Figure S2. UV spectra of AS1411-N6 and AS1411 in presence of 10 mM of LiCaCo buffer supplemented with 140 mM of KCl, at 25°C and 95 °C.

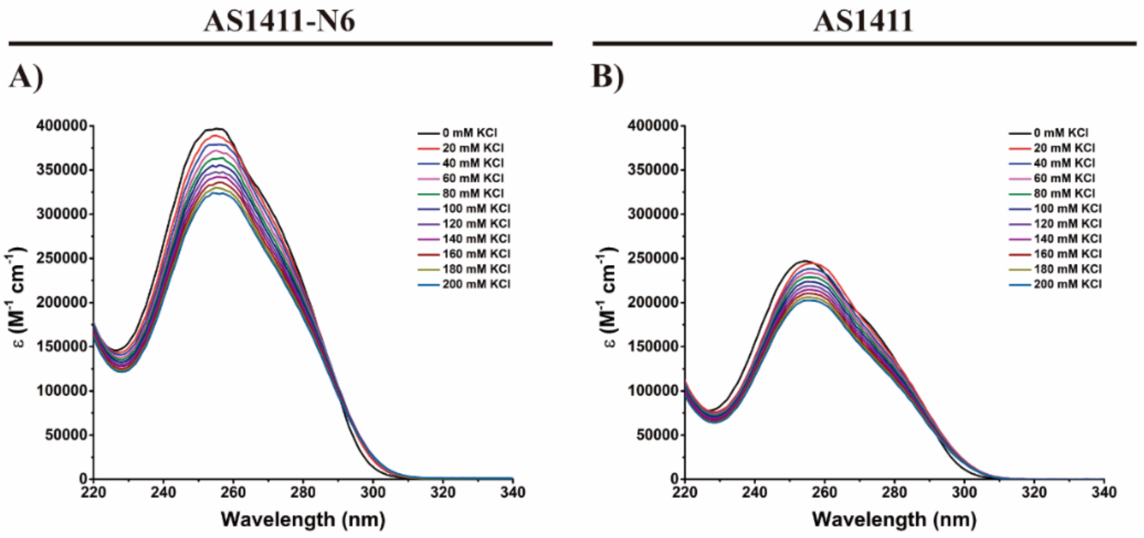

Figure S3. UV spectra of AS1411-N6 (A) and AS1411 (B) in with increasing amounts of KCl.

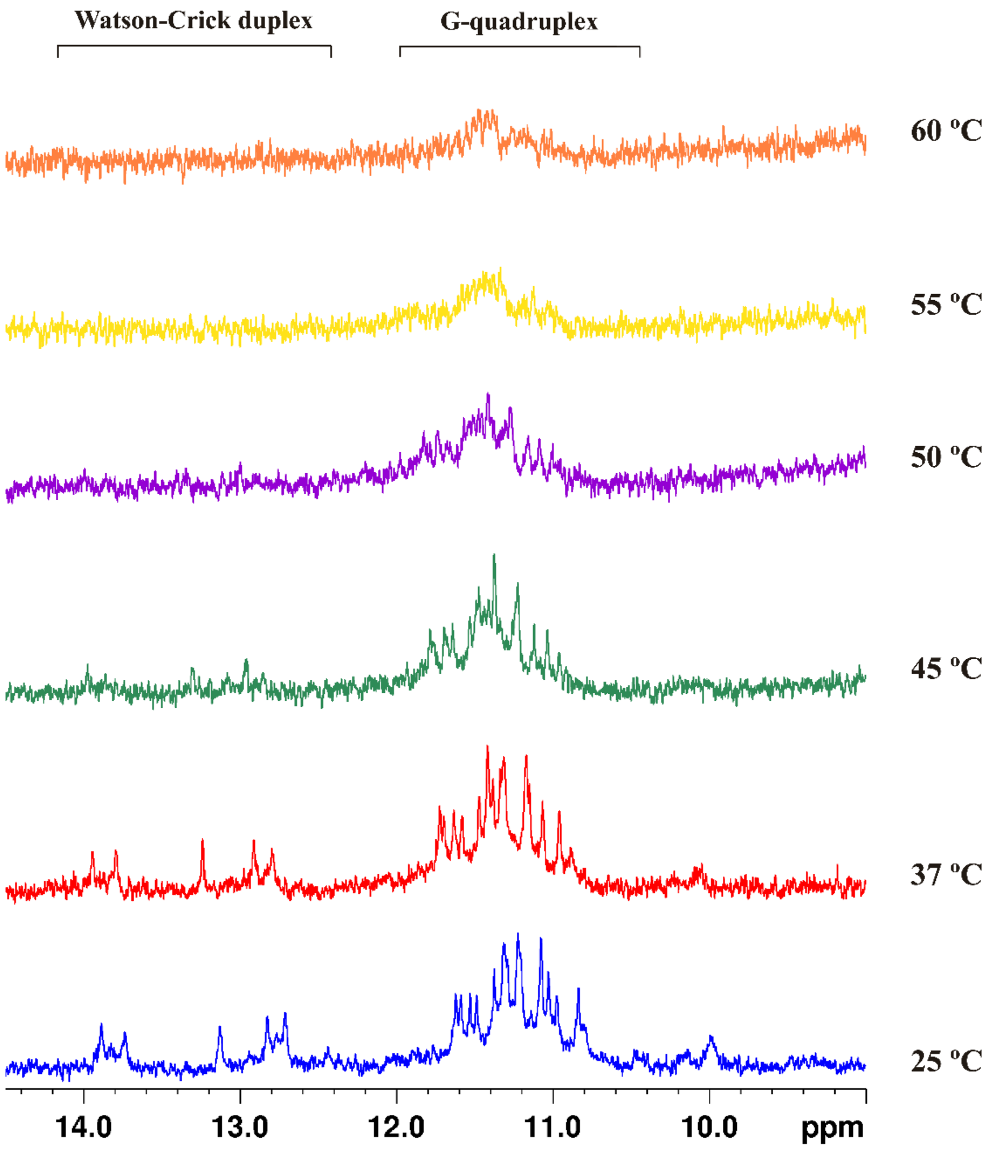

Figure S4. Study of temperature effect in AS1411-N6 G4 structure by ^{1}H NMR spectroscopy. It is evidenced by a superior temperature resistance of the G4 portion instead of the WC duplex. At 45 ºC duplex signal intensities are residual, and thus can be concluded that up this temperature duplex are disrupted, however, G4 maintains.

Table S2. ΔT_m of oligonucleotides in presence of commercial ligands by FRET melting assay

**Aptamer sequence**	**Ligand**	**ΔT_m (°C)**				
		**0.5 eq.**	**1 eq.**	**2 eq.**	**3 eq.**	**4 eq.**
AS1411-N6	PhenDC3	1.47 ± 1.05	6.39 ± 0.80	7.74 ± 0.48	16.19 ± 4.18	22.89 ± 1.49
	PDS	0.29 ± 0.12	1.08 ± 0.15	0.88 ± 0.31	1.65 ± 0.29	2.35 ± 0.44
	BRACO-19	0.03 ± 0.28	1.56 ± 0.18	2.29 ± 1.04	3.36 ± 0.35	3.73 ± 0.09
	TMPyP4	4.22 ± 0.92	11.08 ± 1.98	21.49 ± 0.61	26.77 ± 0.16	28.94 ± 0.23
	360A	6.43 ± 2.094	12.90 ± 1.69	16.10 ± 0.97	18.74 ± 0.50	20.16 ± 0.41
AS1411	PhenDC3	-1.47 ± 0.98	-3.00 ± 1.18	8.66 ± 3.79	18.55 ± 2.03	24.77 ± 2.56
	PDS	-1.65 ± 0.63	-1.88 ± 0.64	-2.14 ± 0.41	-1.51 ± 0.88	-0.75 ± 1.02
	BRACO-19	-0.1 ± 1.25	0.31 ± 0.87	1.94 ± 1.79	7.42 ± 0.92	8.99 ± 0.79
	TMPyP4	6.05 ± 1.74	12.69 ± 0.87	17.13 ± 1.15	20.21 ± 1.19	21.75 ± 1.08
	360A	-2.20 ± 0.94	-2.08 ± 1.08	5.06 ± 2.55	18.07 ± 1.80	19.28 ± 0.99

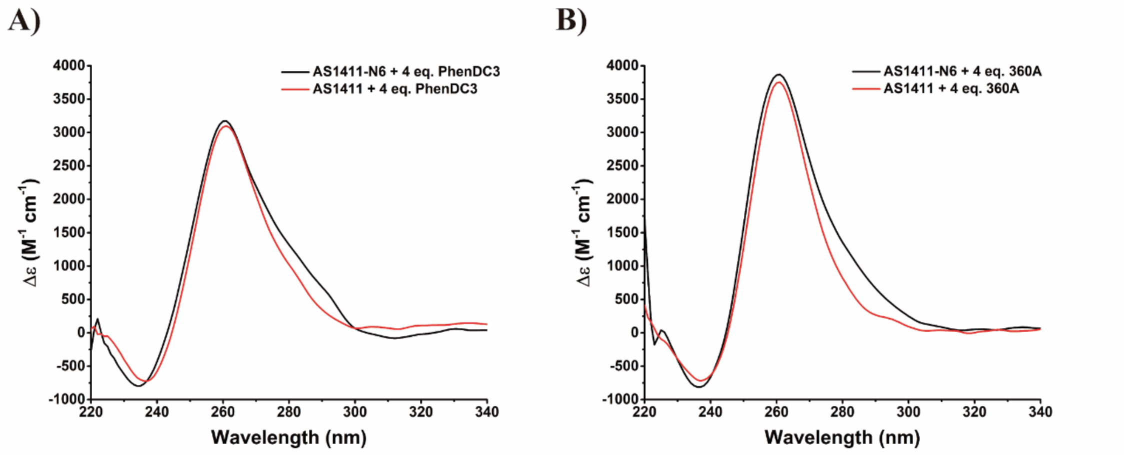

Figure S5. Comparison of the profile of both sequences due to the effect of high concentrations of ligands, namely, PhenDC3 and 360A at 4 molar equivalents. At this concentration, AS1411-N6 seems to disrupt the duplex portion, and the shape of the spectrum is similar to de unmodified aptamer (AS1411).

Table S3. T_m of AS1411-N6 and AS1411 in presence of PhenDC3, 360A, and TMPyP4 by CD melting experiment

**[Ligand] (eq.)**	**ΔT_m (ºC)**					
	**PhenDC3**		**TMPyP4**		**360A**	
	**AS1411-N6**	**AS1411**	**AS1411-N6**	**AS1411**	**AS1411-N6**	**AS1411**
**0.25**	3.10	2.14	1.13	0.74	2.83	0.32
**0.5**	5.30	3.59	4.04	1.80	5.56	1.33
**1**	8.20	6.48	8.31	3.44	9.88	3.81
**2**	17.90	13.64	17.39	9.17	20.83	13.63
**3**	> 30	25.16	21.63	16.15	33.58	25.15
**4**	> 30	> 30	22.62	25.23	> 30	> 30

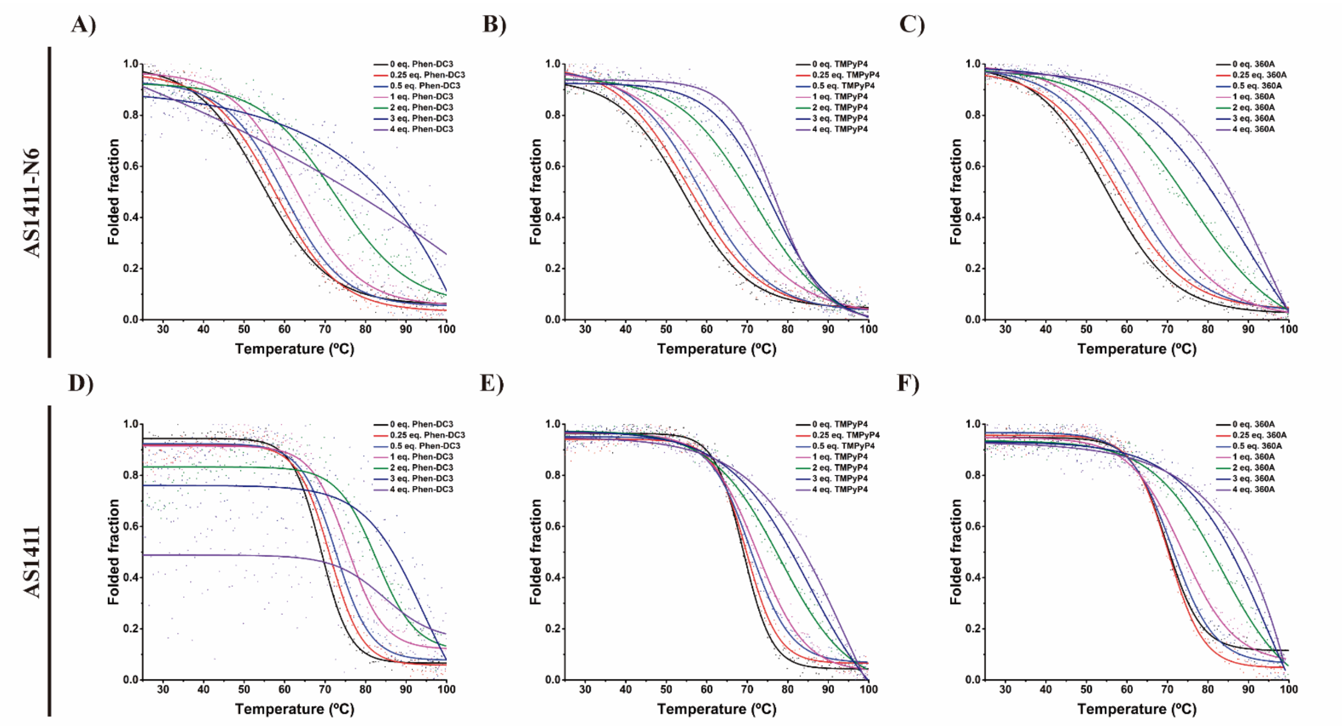

Figure S6. CD-melting curves of aptamers in presence of several ligands at several concentrations. It was observed for all ligands a stabilizer effect in G4 structure.

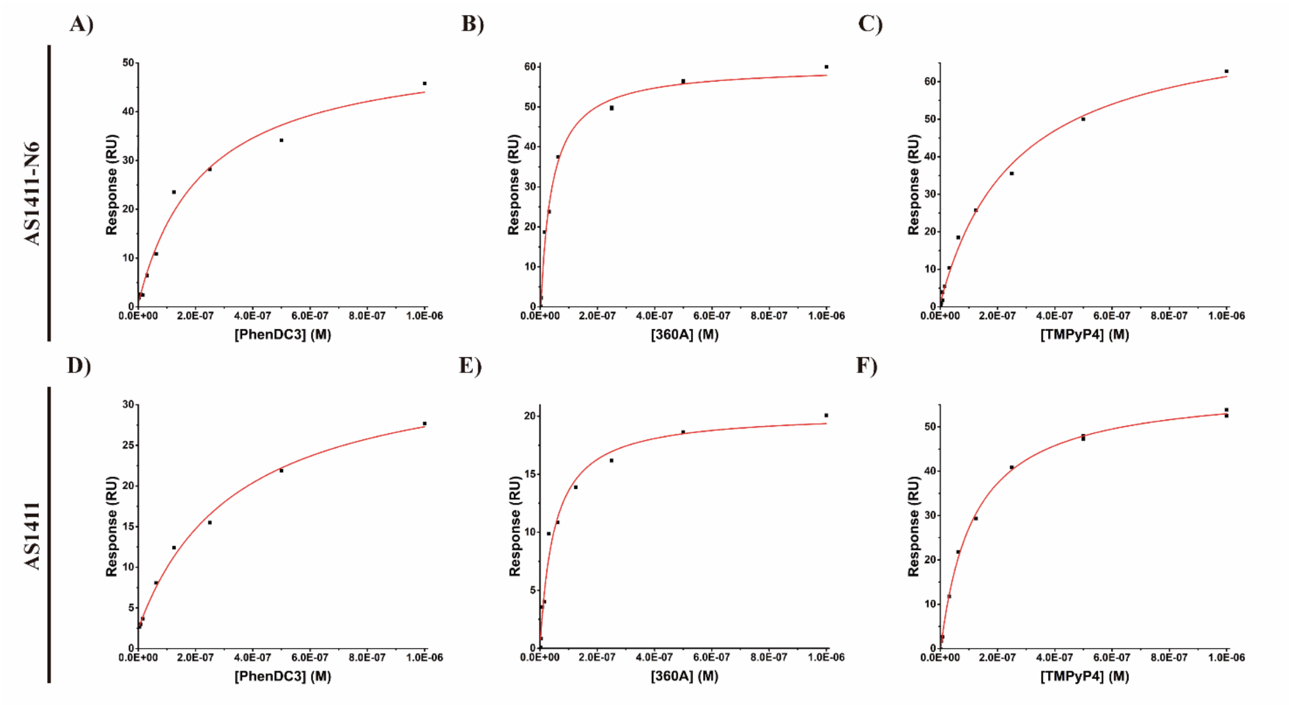

Figure S7. Equilibrium binding curves of AS1411-N6 and AS1411 upon addition of ligands. K_D values were determined by fitting equilibrium binding data using a one-site saturation binding model and listed in Table 3.

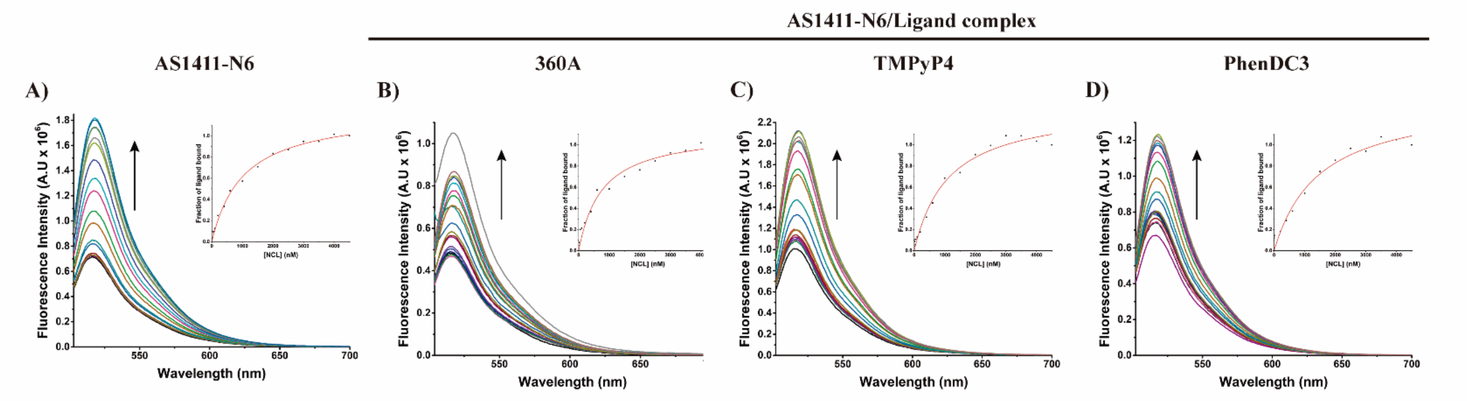

Figure S8. Fluorescence spectra and saturation binding plot analysis of AS1411-N6 and AS1411-N6/ligand complex upon NCL RBD 1,2 titration. The apparent equilibrium dissociation constant (K_D) was obtained through of application of one-site saturation binding function.
